# P-1796. Uptake, Utilization, and Satisfaction of a Mobile Application Decision Support Tool to Enhance Antimicrobial Stewardship in Colorado

**DOI:** 10.1093/ofid/ofae631.1959

**Published:** 2025-01-29

**Authors:** Joana Dimo, Matthew J Weber, Matthew Miller, Lauren Biehle, Michael J Bozzella, Christopher A Czaja, Leigh Anne Bakel, Timothy C Jenkins, Sarah K Parker

**Affiliations:** University of Colorado/Children's Hospital Colorado, Denver, Colorado; University of Colorado/Children's Hospital Colorado, Denver, Colorado; Children's Hospital Colorado, Aurora, Colorado; Colorado Department of Public Health and Environment, Denver, Colorado; Children's Hospital Colorado, Aurora, Colorado; Colorado Department of Public Health and Environment, Denver, Colorado; University of Colorado/Children's Hospital Colorado, Denver, Colorado; Denver Health, Denver, Colorado; University of Colorado/Children's Hospital Colorado, Denver, Colorado

## Abstract

**Background:**

Several barriers to antimicrobial stewardship (ASP) in various Colorado hospitals were previously identified, including lack of clinical pathways, antibiotic dosing knowledge in neonatal and pediatric populations, and understanding of stewardship methodologies. We sought to mitigate barriers to robust ASP in Colorado hospitals through creation of a mobile application decision support system, Firstline (www.Firstline.org). Our objective is to describe the uptake, usage patterns, and satisfaction of Firstline across the state.

Figure 1

**Figure:**
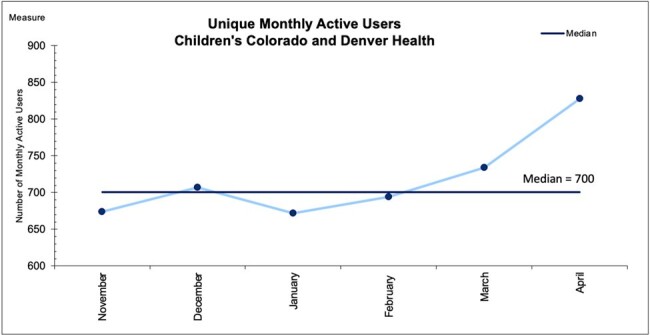

Run chart of monthly active users of the Children’s Colorado and Denver Health Firstline app from beginning of November 2023 through end of April 2024.

**Methods:**

ASP teams at Children’s Hospital Colorado (CHCO) and Denver Health (DH) partnered with Firstline to develop the app with locally curated content including: 1) CHCO and DH clinical pathways, 2) pathogen information, 3) antimicrobial information, and 4) infection prevention and ASP resources. The CHCO platform went live October 10^th^, 2023, and DH on October 31^st^, 2023. Firstline provided analytic data on the number of users and usage patterns. The CHCO Physician Relations team and the Colorado Department of Regulatory Agencies (DORA) Professional and Occupational Licenses Information Marketplace provided Practitioner data. Feedback on the app was obtained via a REDCap survey distributed through Firstline.

Figure 2

**Figure:**
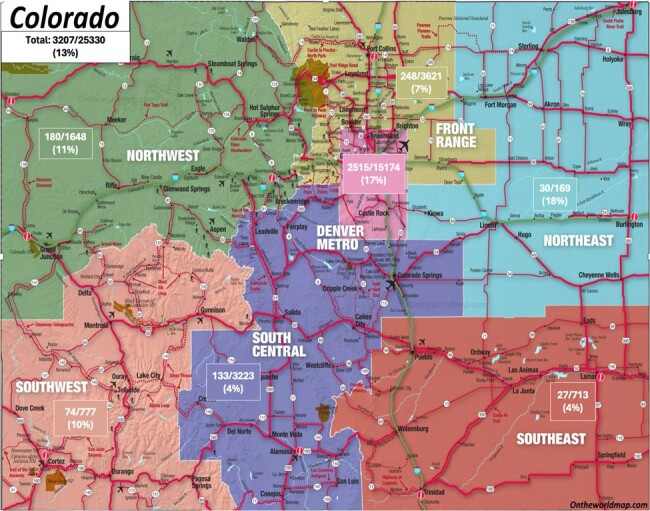

Run chart of monthly active users of the Children’s Colorado and Denver Health Firstline app from beginning of November 2023 through end of April 2024.

**Results:**

A total of 3,287 unique mobile users accessed CHCO or DH Firstline from launch through April 2024. There were 718 average monthly active users (Figure 1). In total, 13% of Colorado practitioners from all regions accessed Firstline through April 2024 (Figure 2). Over 60% of survey responders used the app daily or weekly, and found the app very easy to use. Support with antimicrobial choice or dosing was the most common reason for using the app. The majority of survey responders noticed a positive enhancement in antibiotic selection, dosing, and guideline utilization through use of the app. 100% of respondents advocated for ongoing support of Firstline (Table 1).

Table 1

**Figure:**
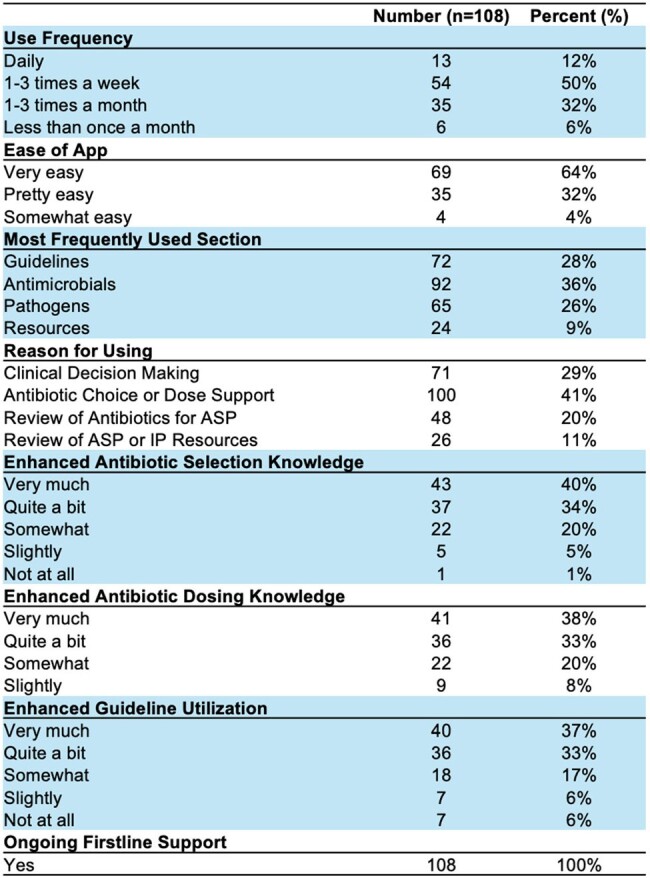

Table of cumulative responses from the REDCap Firstline satisfaction survey. ASP, Antimicrobial Stewardship Program; IP, Infection Prevention.

**Conclusion:**

We disseminated a mobile application support tool to augment ASP in Colorado with positive initial feedback. Widespread dissemination to rural communities in Colorado is ongoing. Future work includes assessing the impact of the app on antimicrobial utilization, guideline adherence, and implementation of priority core CDC elements for ASP.

**Disclosures:**

**Sarah K. Parker, MD**, Colorado Department of Health and Environment: Advisor/Consultant|Pfizer Global Bridges: Grant/Research Support

